# Supplementary material for: Radiomics profiling combined with clinical risk factors for preoperative Lymphatic Metastasis prediction in Colorectal cancer: A multicenter study
Source: PLoS One. 2026 Jan 16;21(1):e0340352. doi: 10.1371/journal.pone.0340352 (PMC12810846; doi:10.1371/journal.pone.0340352)
Supplement: S1 Table — (DOC) [file pone.0340352.s001.doc]

| **Clinical factors** | **LNM- (N = 210)** | **LNM+ (N = 139)** | ***p*** Value |
| --- | --- | --- | --- |
| **Age (year)** | 65.0±11.2 | 63.8±11.3 | 0.300 |
| **Sex (N)** |  |  | 0.512 |
| Male | 128 | 79 |  |
| Female | 82 | 60 |  |
| **Swollen lymph nodes** |  |  | 0.001* |
| YES | 132 | 60 |  |
| NO | 78 | 79 |  |
| **AFP ug/L** | 2.45(1.9,3.5) | 2.6(1.9,3.4) | 0.795 |
| **CEA ug/L** |  |  | 0.01* |
| ＜5.0 | 139 | 72 |  |
| ≥5.0 | 71 | 67 |  |
| **CA199 U/ml** |  |  | 0.028* |
| ＜37.0 | 188 | 112 |  |
| ≥37.0 | 22 | 27 |  |
| Normally distributed factors are expressed using means ± standard deviations; non-normally distributed factors are expressed as medians (interquartile ranges) *With a p < 0.050 | | | |
