# Supplementary material for: Radiomics profiling combined with clinical risk factors for preoperative Lymphatic Metastasis prediction in Colorectal cancer: A multicenter study
Source: PLoS One. 2026 Jan 16;21(1):e0340352. doi: 10.1371/journal.pone.0340352 (PMC12810846; doi:10.1371/journal.pone.0340352)
Supplement: S2 Table — (DOC) [file pone.0340352.s002.doc]

| **Classification** | **Factor** | **short title** |
| --- | --- | --- |
| **Clinical factors** |  |  |
|  | CA199 | Feature 6 |
|  | CEA | Feature 7 |
|  | Swollen_lymph_nodes | Feature 8 |
| **3D(R) factors** |  |  |
|  | original_shape_Compactness | Feature 0 |
|  | log-sigma-3-mm-3D_glszm_GrayLevelNonUniformity | Feature 3 |
|  | wavelet-HLH_glcm_InverseVariance1 | Feature 4 |
|  | 5:wavelet-LHH_glszm_SmallAreaHighGrayLevelEmphasis | Feature 5 |
| **3D(C) factors** |  |  |
|  | log-sigma-3-mm-3D_gldm_SmallDependenceHighGrayLevelEmphasis | Feature 1 |
|  | log-sigma-3-mm-3D_glszm_LowGrayLevelZoneEmphasis | Feature 2 |
