# Supplementary material for: Radiomics profiling combined with clinical risk factors for preoperative Lymphatic Metastasis prediction in Colorectal cancer: A multicenter study
Source: PLoS One. 2026 Jan 16;21(1):e0340352. doi: 10.1371/journal.pone.0340352 (PMC12810846; doi:10.1371/journal.pone.0340352)
Supplement: S3 Table — (DOC) [file pone.0340352.s003.doc]

|  | | **Delong test** |
| --- | --- | --- |
| **Training dataset** | |  |
| Model3D(R) vs. Model3D(R+C) | | 0.026* |
| ModelC vs. Model3D(R+C) | | 0.031* |
| ModelC vs. ModelC3D(R+C) | | <0.001* |
| Model3D(R+C) vs. ModelC3D(R+C) | | <0.001* |
| **Validation dataset** | |  |
| Model3D(R) vs. Model3D(R+C) | | 0.179 |
| ModelC vs. Model3D(R+C) | | 0.04* |
| ModelC vs. ModelC3D(R+C) | | <0.001* |
| Model3D(R+C) vs. ModelC3D(R+C) | 0.025* | |
| Data are presented as P values. * (P < 0.050 is significant) | | |
