# Supplementary material for: Radiomics profiling combined with clinical risk factors for preoperative Lymphatic Metastasis prediction in Colorectal cancer: A multicenter study
Source: PLoS One. 2026 Jan 16;21(1):e0340352. doi: 10.1371/journal.pone.0340352 (PMC12810846; doi:10.1371/journal.pone.0340352)
Supplement: S4 Table — (DOC) [file pone.0340352.s004.doc]

|  | **AUC** | **sensitivity** | **specifity** |
| --- | --- | --- | --- |
| **Training dataset** |  |  |  |
| Model3D(R) | 0.696 | 0.564 | 0.720 |
| Model3D(R+C) | 0.733 | 0.827 | 0.511 |
| ModelC | 0.635 | 0.655 | 0.593 |
| ModelC3D(R+C) | 0.858 | 0.764 | 0.835 |
| **Validation dataset** |  |  |  |
| Model3D(R) | 0.563 | 0.333 | 0.875 |
| Model3D(R+C) | 0.641 | 0.593 | 0.667 |
| ModelC | 0.589 | 0.444 | 0.583 |
| ModelC3D(R+C) | 0.833 | 0.556 | 0.917 |
