# Supplementary material for: Radiomics profiling combined with clinical risk factors for preoperative Lymphatic Metastasis prediction in Colorectal cancer: A multicenter study
Source: PLoS One. 2026 Jan 16;21(1):e0340352. doi: 10.1371/journal.pone.0340352 (PMC12810846; doi:10.1371/journal.pone.0340352)
Supplement: Attachment — Submitted filename: Comment to the author1.docx [file pone.0340352.s010.docx]

This study presents an intriguing investigation aimed at preoperatively predicting LNM metastasis in CRC patients by using a model consisting of a combination of radiomics and clinical risk factors. The study's innovative approach holds some clinical value, shedding light on the prospect of utilizing radiomics for predicting LNM metastasis in CRC patients. However, in my opinion, the worrisome aspects of this manuscript are as follows:

1) The proportion of patients diagnosed with rectal cancer was unclear, and the tumor deposits were not shown in Table 1, which could affect the N stage of rectal cancer.

2) In the patient selection section, you enrolled the patients who had postoperative pathology reports confirmed CRC, but you did not clarify the type of histopathology. How did you cope with this?

3) The arrows used in Figure 1 were less accurate.

4) You stated that “The images obtained using this tool were also evaluated and confirmed by 2 senior imaging physicians, and their reproducibility was similarly satisfactory”, how did you ensure the reproducibility of your data? It would have been important to examine the variability of results concerning the ROI variation.

5) How did you control colorectal movement during image acquisition? Have you applied some form of motion correction in image post-processing?

6) The graphical abstract showed several methods for model building including SVM, Radom Forest, Decision Tree, etc, have you compared the differences among the methods for model building? That is to say, why did you choose SVM for model building?

7) English is not always clear, English editing is strongly suggested.
