# Supplementary material for: Radiomics profiling combined with clinical risk factors for preoperative Lymphatic Metastasis prediction in Colorectal cancer: A multicenter study
Source: PLoS One. 2026 Jan 16;21(1):e0340352. doi: 10.1371/journal.pone.0340352 (PMC12810846; doi:10.1371/journal.pone.0340352)
Supplement: Attachment — Submitted filename: Response to Reviewers.docx [file pone.0340352.s012.docx]

**Reviewer #1**

1) The proportion of patients diagnosed with rectal cancer was unclear, and the tumor deposits were not shown in Table 1, which could affect the N stage of rectal cancer.

Thank you for your feedback regarding the proportion of patients with rectal cancer and the consideration of tumor deposits.

Proportion of Patients with Rectal Cancer: We have specified that approximately 10% of patients in our study were diagnosed with rectal cancer in the revised manuscript.

Impact of Tumor Deposits: In this study, we primarily utilized lymph node metastasis status from pathology reports to predict preoperative lymph node metastasis. Thus, tumor deposits had minimal impact on our analysis and findings. Additionally, since the majority of cases showed no significant tumor deposits in pathology reports, this variable was not emphasized in Table 1 (Rows 186-190).

We hope this response addresses your queries, and we thank you for your valuable comments.

2) In the patient selection section, you enrolled the patients who had postoperative pathology reports confirmed CRC, but you did not clarify the type of histopathology. How did you cope with this?

We appreciate the reviewers' thorough review of our manuscript. In this study, adenocarcinoma and mucinous adenocarcinoma were identified as the predominant histologic subtypes of colorectal cancer. For patient selection, we specifically included cases of these two subtypes, thus limiting our discussion of other histologic types in the manuscript. This focus ensures relevance and validity in our results, particularly regarding the preoperative prediction of lymph node metastasis (LNM). We appreciate this suggestion and will incorporate relevant annotations in the revised manuscript to enhance clarity and transparency.

3) The arrows used in Figure 1 were less accurate.

We appreciate the feedback regarding the accuracy of the arrows in Figure 1. Figure 1 has been revised to enhance the accuracy and placement of the arrows, ensuring they clearly indicate the intended processes and relationships. Thank you once again for your valuable insights.

4) You stated that “The images obtained using this tool were also evaluated and confirmed by 2 senior imaging physicians, and their reproducibility was similarly satisfactory”, how did you ensure the reproducibility of your data? It would have been important to examine the variability of results concerning the ROI variation.

Response to Reviewer: We thank the reviewers for their valuable comments regarding reproducibility and the importance of ROI variability. Recognizing this, we implemented several steps to validate the reliability of our data. Two senior imaging physicians annotated the colorectal regions in a subset of cases following a standardized protocol. To assess reproducibility, we calculated the intra-group correlation coefficient (ICC) between physician-annotated images and those automatically labeled by our tool, yielding an ICC of 0.994, which demonstrates high concordance. Furthermore, we conducted multiple automated segmentations on the same patient images, achieving an ICC of 0.989, indicating strong reproducibility in image segmentation by the tool.

While these results confirm our study's reproducibility, we acknowledge that further analysis of ROI variability could enhance the robustness of our findings. Future studies will incorporate a more detailed examination of ROI variability to optimize reproducibility further. We appreciate this insightful suggestion and will include these details in the revised manuscript. (Lines 161-167)

5) How did you control colorectal movement during image acquisition? Have you applied some form of motion correction in image post-processing?

Thank you for your insightful question regarding control of bowel movement and motion correction.

Control of bowel motion: During image acquisition, a standardised scanning protocol was followed to minimise bowel movement. Patients were instructed to remain still and in some cases were asked to hold their breath after a deep inhalation to further reduce motion artefacts associated with respiratory movement.

Motion correction in post-processing: We did not apply any specific motion correction algorithms during image post-processing. The imaging sequences and acquisition parameters were optimised to limit motion artefacts and ensure adequate image quality for radiomic analysis. Given the standardised scanning protocol and patient guidance, motion-related issues were minimal in our dataset and no further motion correction was required.

We hope this answers your question and appreciate the opportunity to clarify our methodology.

6) The graphical abstract showed several methods for model building including SVM, Radom Forest, Decision Tree, etc, have you compared the differences among the methods for model building? That is to say, why did you choose SVM for model building?

Response to Reviewer: Thank you for your question regarding the selection of modeling methods. In this study, we initially assessed five machine learning algorithms, including support vector machines (SVM), random forests, and decision trees, to determine the most suitable approach for predicting lymph node metastasis (LNM) in colorectal cancer. We evaluated these methods based on key performance metrics—such as area under the curve (AUC), accuracy, sensitivity, and specificity—to comprehensively assess their predictive capabilities. The Discussion section (lines 312–313) details the performance comparison of these five models, supplemented by Figure 1, which graphically illustrates the AUC differences, providing a visual comparison of model performance on the dataset.

Rationale for Choosing SVM: Among the algorithms tested, SVM consistently demonstrated superior performance across key metrics, particularly AUC. SVM's ability to effectively handle high-dimensional data and complex imaging histologic features was a critical factor in our selection. Additionally, SVM’s low tendency for overfitting and robust classification boundary management further support its suitability for this study.

7) English is not always clear, English editing is strongly suggested.

Response to Reviewer: Thank you for your suggestion regarding the clarity of the English. We have thoroughly reviewed the manuscript and arranged for professional editing to enhance readability and language quality. The revised text has been carefully refined for clarity and precision. We hope these revisions will contribute to improved overall clarity of the manuscript. Once again, we appreciate your valuable feedback.

**Reviewer #2**

MeSH BASED KEYWORDS ONLY NEED SOME AMENDMENTS, LYMPHNODE METASTASIS NEED STO BE CORRECTED AND RADIOMICS CHARACTERIZATION OF TUMOURS NEED TO BE REDONE

Thank you very much for your constructive comments. In response, we have revised the keywords to align with MeSH standards. Specifically, the term "lymph node metastasis" has been corrected, and all radiological features of the tumor have been rewritten accordingly. In the revised version, all corrected parts are highlighted in red font for clarity.

**Reviewer #3**

1. Perhaps there should be more explanation in the text of what is meant by the colorectal region, what are its boundaries. How were the structures segmented in colorectal ROI (lymph nodes, intestine, paracolitic tissue)?

Thank you very much for your valuable feedback. We have revised the manuscript to provide a clearer definition of the colorectal region and to detail the segmentation process for the structures within this region of interest (ROI). Below are the specific changes made:

We have added an introduction to the colon region in the Image Acquisition and 3D Segmentation section of the text: The Colorectal Region in this study refers to the anatomical region from the cecum to the rectum and includes the colon, rectum and surrounding paracolic tissue. The boundaries of this region are defined by key anatomical landmarks, including the segments of the colon and the upper and lower ends of the rectum.

In this study, the segmentation of the colorectal region of interest (ROI) focused specifically on delineating the tumor area, excluding structures such as lymph nodes, the intestinal wall, and paracolic tissue. We employed a manual segmentation approach using ITK-SNAP software, where the primary goal was to accurately outline the tumor within the colorectal region while avoiding the inclusion of other structures.

As colorectal tumors are generally well-defined in venous-phase CT images, this manual segmentation method proved to be reliable and reproducible. To ensure the accuracy and consistency of the segmentation, all outlined ROIs were reviewed and confirmed by two senior gastrointestinal radiologists. This process ensured that only the tumor region was included and that other structures, such as lymph nodes or normal intestinal tissues, were not part of the segmented tumor ROI.And we also go into more detail in the manuscript.

We hope this explanation clarifies the segmentation approach used in our study. If you have any further suggestions or questions, we would be happy to address them.

2. Colorectal cancer is a broad term and includes tumors of various parts of the colon and rectum with its anatomical features. Did this fact somehow influence the segmentation of the area of interest? If so, how? If not, why?

Thank you for raising this important point. We acknowledge that colorectal cancer encompasses tumors located in different anatomical regions of the colon and rectum, each with unique characteristics. However, in our study, these anatomical variations did not significantly affect the segmentation of the region of interest (ROI).

To address this, our segmentation method was standardized to focus on the tumor boundaries, regardless of its location within the colorectal region. Since we utilized contrast-enhanced venous phase CT images, tumors in different anatomical locations are generally well-defined, which allowed us to apply a consistent manual segmentation approach across all cases. By concentrating on the well-defined tumor boundaries, we ensured reliable segmentation of both colon and rectal tumors.

3. There is missed reference - line 73.

Thank you for pointing out the omitted reference on line 73. We have now added the appropriate reference to the revised manuscript. The citation has been added to the relevant section to ensure completeness and proper attribution. Thank you for your attention to this detail.

4. Words are merged in some places in the text.

Thank you for your careful review and for pointing out that some words have been merged in the text. We have carefully reviewed the manuscript and corrected all instances where words were unintentionally merged. We have also carefully checked the formatting to ensure clear spacing and readability throughout the document.

We appreciate your attention to detail in helping us improve the clarity of our manuscript.
