# Supplementary material for: Radiomics profiling combined with clinical risk factors for preoperative Lymphatic Metastasis prediction in Colorectal cancer: A multicenter study
Source: PLoS One. 2026 Jan 16;21(1):e0340352. doi: 10.1371/journal.pone.0340352 (PMC12810846; doi:10.1371/journal.pone.0340352)
Supplement: Attachment — Submitted filename: Response to Reviewers.doc [file pone.0340352.s013.doc]

Reviewer #4: 
1) Please comment if any pre-processing of the CT images was done to ensure that the validation data was harmonized with the training data.
Thank you for your valuable comment. In response to your query regarding the pre-processing of the CT images, we have taken several steps to ensure that the validation data is harmonized with the training data.

1.CT Image Selection: We selected the venous-phase images for both the training and validation datasets, ensuring consistency in the imaging phase across allcases.

2.Image Resizing and Dimensions: All CT images were resized to a consistentdimension of 512x512 pixels to ensure uniformity in image resolution.

3.Image Slice Thickness: A slice thickness of 5mm was applied to all images, both in the training and validation datasets, to maintain uniformity in the 3D reconstruction of the tumors.

4.Image Windowing: During the image segmentation process, we set the Level and Window values to 100 and 300, respectively, for all images. This helped standardize the contrast and brightness of the CT images across the datasets.

These pre-processing steps were applied uniformly to both the training and validation datasets to ensure that the data used for model training and validation were harmonized and comparable. (Lines 133-144)

2) Please comment on the combined model's sensitivity to potential segmentation errors of the tumor and colorectal regions with the automated tool to different noise levels in the images.
Thank you for your thoughtful comment. In our study, we used both manual and automated segmentation techniques to delineate the tumor and colorectal regions, ensuring the accuracy of the tumor area without including surrounding structures such as lymph nodes.

1.Tumor Segmentation: The tumor region was manually segmented using ITK-SNAP software, with venous-phase CT images guiding the process. Given the well-defined appearance of colorectal tumors in CT images, this manual segmentation approach was both effective and reproducible. To ensure segmentation accuracy, two senior gastrointestinal radiologists reviewed and confirmed all manually segmented areas, ensuring no inclusion of non-tumor structures such as lymph nodes or paracolic tissue.

2.Colorectal Region Segmentation: The colorectal region, which includes the colon, rectum, and surrounding paracolic tissue, was segmented using the automated Total Segmentator tool. This tool was chosen for its ability to capture the full extent of the colorectal region, and the resulting segmentations were reviewed and confirmed by two senior imaging physicians. The high intra-group correlation coefficient (ICC) of 0.994 between physician-annotated images and tool-generated segmentations demonstrates strong agreement and high reproducibility.

3.Sensitivity to Segmentation Errors and Noise: While both manual and automated segmentation techniques were rigorously validated for accuracy and reproducibility, we recognize that the presence of noise or segmentation errors could potentially affect model performance. Given that segmentation of the colorectal region is a challenging task, particularly with respect to surrounding tissue structures, we acknowledge that noise and segmentation errors may impact the radiomic features extracted from the images. However, the high ICC values and reproducibility achieved through both manual and automated segmentation methodssuggest that the model is relatively robust to minor variations in segmentation accuracy.

In future work, it would be beneficial to evaluate the sensitivity of the combined model to segmentation errors and image noise through perturbation tests or adversarial noise simulations, which would further assess its robustness under different conditions.

3) Please mention how the regularization parameter for LASSO was chosen for the feature selection of the radiomics features.
Thank you for your valuable comment. In our study, the regularization parameter for LASSO (Least Absolute Shrinkage and Selection Operator) was chosen through a systematic process to optimize the feature selection from the radiomics features.

1. LASSO Regularization Parameter Selection: The optimal value for the regularization parameter (lambda, λ) was determined using cross-validation. Specifically, a 10-fold cross-validation procedure was applied to the training dataset. We tested a range of lambda values, and the one that minimized the mean square error (MSE) across the folds was selected as the optimal parameter. This approach ensures that the model avoids both overfitting (by choosing a lambda thatis too small) and underfitting (by selecting a lambda that is too large).
2. Model Tuning: By performing this process, we ensured that the LASSO method selected the most relevant features while minimizing the inclusion of irrelevant or redundant features, thereby improving the model's generalizability and predictive performance.

In future work, we plan to explore other feature selection methods and furtherfine-tune the LASSO regularization parameter to improve model accuracy and robustness.

3. 4) It would be interesting to compare the proposed combined model against a basic metric such as the mean, standard deviation, and size of the tumor ROI.
   Thank you for your suggestion to compare the proposed combined model with basic metrics such as the mean, standard deviation, and size of the tumor ROI. I have conducted the comparison, and the results are as follows:

A comparison of the combined model with the models based on mean, standard deviation, and tumor ROI size shows that the combined model demonstrates superior performance in terms of AUC on both datasets:

Training set (AUC = 0.858 vs. 0.575 vs. 0.541 vs. 0.579)

Validation set (AUC = 0.833 vs. 0.426 vs. 0.467 vs. 0.444)

These results clearly highlight that the combined model significantly outperforms the models that are built directly from basic parameters.(Lines 258-269)

5) Have the authors considered using a deep-learning neural network for classifying the radiomics features?

Thank you for your insightful comment. We appreciate your suggestion regarding the potential use of deep learning for classifying radiomics features.

1. Current Approach and Rationale: In our study, we focused on using traditional machine learning methods, specifically the combined model with LASSO for feature selection and a subsequent classifier, due to their proven interpretability, efficiency, and established success in radiomics-based research. These methods allow for clearer insights into which features contribute most to model performance, which is crucial in medical imaging applications where interpretabilityis key for clinical decision-making.
2. Deep Learning Consideration: While deep learning approaches, especially convolutional neural networks (CNNs), have shown great promise in medical image analysis, they typically require large labeled datasets to achieve high performance. In our study, the dataset size and feature extraction approach led us tofocus on a more interpretable traditional machine learning method. However, weacknowledge that deep learning could potentially further enhance the model’s predictive power, especially in capturing complex patterns within radiomics data.

Future Work: Future Work: Given the rapid advances in deep learning and its increasing success in medical imaging, we plan to explore deep learning techniques in future work. This could involve the use of neural networks for directly analyzing image data or applying deep learning-based feature selection methods before classification. We believe this would complement our existing model and may provide additional predictive power in more complex datasets. As noted in the last section of the manuscript (lines 368-370), we state that "future studies may employ deep learning techniques for radiomics feature extraction, eliminating the need for manual feature design and enabling the capture of more complex data relationships." We will consider this approach in future studies, where a larger dataset or different modalities might be available to leverage the advantages of deep learning techniques.

Reviewer #5

1) What was the false positive rate of the combined model in the validation cohort?

Thank you for your insightful comment.

The false positive rate (FPR) of the combined model in the validation cohort is 0.0833, which indicates that approximately 8.33% of the negative cases in the validation set were incorrectly classified as positive.

As detailed in Supplementary Table 4, we have provided the following key performance metrics for the validation cohort:

AUC: 0.833

Sensitivity: 0.556

Specificity: 0.917

These parameters collectively evaluate the model's performance in predicting preoperative lymph node metastasis (LNM) in CRC patients. The FPR, alongsideAUC, sensitivity, and specificity, provides a comprehensive assessment of the model's diagnostic capability. The false positive rate (FPR) is an essential metric to evaluate the reliability of the model in clinical practice. In our study, the combined model achieved a relatively low FPR of 8.33% in the validation cohort, indicating that the model performed well in distinguishing tumor from non-tumor cases. A low false positive rate is crucial in clinical decision-making, as it helps to reduce unnecessary interventions and avoids the over-diagnosis of tumors, which could lead to unnecessary treatments or tests. Although there were some misclassifications, the model demonstrated a good balance between sensitivity, specificity, and FPR, contributing to its robust performance in predicting preoperative lymph node metastasis (LNM) in CRC patients.(Lines 349-357)

2) Could the inclusion of MRI or PET features further improve prediction?

Thank you for your excellent suggestion. The inclusion of additional imaging modalities such as MRI or PET features could indeed potentially improve the model's prediction accuracy.

1. MRI Features: MRI has been shown to provide superior soft tissue contrast, particularly in detecting lymph node metastases and characterizing tumors with high precision. MRI features, such as texture, shape, and diffusion-weighted imaging (DWI) parameters, could offer complementary information to the radiomic features extracted from CT scans. Integrating MRI features could potentially enhance the model's ability to identify regional lymphatic metastases (LNM),especially in challenging cases where CT images alone might be less conclusive.

2. PET Features: PET imaging is widely recognized for its ability to assess metabolic activity, which is crucial for detecting active tumor regions and lymph node metastases. The inclusion of PET-derived features, such as maximum standardized uptake value (SUVmax), could improve the prediction by providing functional information on the tumor's metabolic activity, which may be a key differentiator between benign and malignant lesions. PET features have been shown to contribute significantly to the accurate staging of cancers, and their integration with radiomics and clinical factors could further refine the model's predictive power.

However, it is important to note that PET is still relatively underutilized in thepreoperative assessment of colorectal cancer patients. PET scanners are not widely available, and only certain hospitals—typically larger tertiary hospitals—are equipped with PET facilities. This makes the inclusion of PET features in routine clinical practice challenging, especially in resource-limited settings. As such,the integration of PET features may need to be limited to specialized centers with the necessary infrastructure.

3. Potential Improvements in Prediction: Combining CT, MRI, and PET features could provide a more comprehensive view of the tumor's structural, functional, and metabolic characteristics, thereby improving the model's performance in predicting LNM. However, the integration of multiple imaging modalities requires advanced techniques in feature extraction, data fusion, and model training. Future studies could explore these multimodal approaches to enhance the accuracy and robustness of preoperative prediction models.

3) Was the model performance statistically compared between training and validation sets?

Thank you for your insightful question. Yes, the performance of all the models was statistically compared between the training and validation sets.

1. AUC Curve Comparison: As shown in Figure 2, we performed a direct comparison of the AUC (Area Under the Curve) for the models trained on the training set versus those validated on the validation set. This visual comparison highlights the discriminative ability of each model in both datasets, ensuring thatthe models are performing consistently across different cohorts.

2. Statistical Comparison of Key Metrics: Additionally, we provided a detailed comparison of key performance metrics, including AUC, sensitivity, and specificity, between the training and validation sets in Supplementary Table 4. This table allows for a more quantitative assessment of the model's performance, showing the AUC, sensitivity, and specificity values for each model in both sets.

These comparisons confirm that the model's performance was consistent and reliable when tested on unseen data in the validation cohort, providing further validation for its generalizability in clinical applications.

4) The limitations are acknowledged but could include a note about potential image quality variability across centers.

Thank you for the suggestion. We acknowledge the importance of image quality variability across different centers, and we agree that this could be a limitation of our study.

While we focused on utilizing standardized imaging protocols to minimize variability, we recognize that differences in scanner models, image resolution, and acquisition techniques across centers may still introduce some degree of variability in the quality of the images used for radiomic feature extraction. This could affect the consistency and reproducibility of the model’s performance when applied to images from different institutions.

We will revise the limitations section of the manuscript to include a note on this potential issue. Specifically, although the two-center design helps validate the model’s generalizability to some extent, broader validation across multiple centers is necessary for more robust validation and assessment. Image quality variability across centers is another limitation to consider. Although standardized imaging protocols were used, differences in scanner models, image resolution, and acquisition techniques could still introduce variability in the quality of images, which may affect the consistency of feature extraction and model performance across centers. .(Lines 359-365)
