# Supplementary material for: Radiomics profiling combined with clinical risk factors for preoperative Lymphatic Metastasis prediction in Colorectal cancer: A multicenter study
Source: PLoS One. 2026 Jan 16;21(1):e0340352. doi: 10.1371/journal.pone.0340352 (PMC12810846; doi:10.1371/journal.pone.0340352)
Supplement: Attachment — Submitted filename: Response_to_Reviewers_auresp_3.docx [file pone.0340352.s014.docx]

Comment 1: The study used data from two centers, but the external validation set was relatively small (n=57). Could the authors discuss how this may affect the generalizability of their findings and whether larger multicenter validation is planned?

Response: We thank the reviewer for this constructive comment. We agree that the relatively small external validation cohort (n=57) limits the precision of performance estimates and may affect the generalizability of our findings. While the two-center design provides preliminary external evidence, a small external sample can widen confidence intervals for metrics such as AUC and may not adequately reflect inter-institutional heterogeneity in case-mix and imaging quality. As noted in the Discussion (Lines 359–361), we have acknowledged the limitations of the two-center design and agree that broader multicenter validation is needed. In the revised manuscript, we further clarify the impact of the small external cohort and outline our plan for wider validation. At present, we are engaging in research exchanges with multiple centers and hope to collect and utilize multicenter data for external validation in subsequent work.

Changes in the manuscript: We have added the following text to the Discussion (Limitations) to explicitly acknowledge the effect of the small external cohort and our plan for broader validation: “It should be noted that the external validation cohort was small (n=57), which may lead to wider confidence intervals around performance estimates and therefore warrants cautious interpretation. Although the two-center design provides preliminary evidence of generalizability, broader multicenter validation is necessary to better capture heterogeneity across institutions. At present, we are engaging in research exchanges with multiple centers and hope to collect and utilize multicenter data for external validation in subsequent work.”(Lines 378–384).

Comment 2: The model is based solely on venous-phase CT images. Since MRI and PET offer complementary functional and metabolic information, have the authors considered multimodal imaging integration to further enhance predictive accuracy?

Response: We appreciate this insightful suggestion. We focused on venous-phase CT because it is routinely acquired for preoperative staging in CRC and is widely available across centers, supporting clinical feasibility and generalizability. At study inception, we did consider incorporating MRI- and PET-derived features. However, based on clinical practice in CRC, contrast-enhanced CT is the most commonly available modality, and MRI/PET are not uniformly accessible across hospitals, which further constrained multimodal integration in this retrospective two-center cohort. In addition, standardized MRI/PET data were not consistently available, and inter-center protocol variability would have complicated robust integration and increased the risk of overfitting given the modest sample size.Nonetheless, we agree that MRI (e.g., T2-weighted, DWI/ADC) and PET (e.g., SUV-based metrics) can provide complementary functional and metabolic information that may enhance model performance. We are currently engaging in research exchanges with multiple centers with the aim of collecting datasets from CRC patients who have CT, MRI, and PET imaging available, upon which we will explore developing a higher-performing multimodal predictive model.

Changes in the manuscript: Our model was developed using venous-phase CT alone to prioritize availability and clinical practicality. Although we initially considered incorporating MRI/PET features, in routine CRC practice contrast-enhanced CT is most widely accessible, whereas MRI and PET are not uniformly available across hospitals. Nonetheless, MRI (e.g., T2-weighted, DWI/ADC) and PET (e.g., SUV-based metrics) may provide complementary functional and metabolic information that could enhance performance. We are currently engaging in research exchanges with multiple centers with the aim of collecting datasets from CRC patients who have CT, MRI, and PET imaging available, upon which we will explore developing a higher-performing multimodal predictive model.(Lines 295–303).

Comment 3: The combined model showed good discrimination (AUC >0.83), but sensitivity remained moderate (55.6%). How would this impact clinical decision-making, and what thresholds do the authors recommend for balancing false negatives and false positives in practice?

Response: We appreciate the reviewer’s important question. While the model achieved strong overall discrimination (AUC >0.83) and a low false positive rate (FPR) of 8.33% in the validation cohort—indicating high specificity (~91.7%) and reduced risk of over-treatment—the sensitivity at the chosen operating point was moderate (55.6%). This implies a non-negligible risk of false negatives (missed LNM) and highlights that the model should complement, rather than replace, clinical judgment and conventional imaging.

Threshold selection should be tailored to clinical priorities. When the consequence of missing LNM is high (e.g., management would materially change), lowering the decision threshold to increase sensitivity is advisable, accepting a higher FPR. Conversely, when minimizing over-treatment is paramount, a higher threshold can be used to favor specificity. For routine use, a balanced operating point (e.g., near the Youden index) can provide a reasonable trade-off. We recommend that institutions calibrate thresholds to local case-mix and resource considerations, guided by model calibration and decision-curve analysis to maximize net benefit. Probability-based risk stratification (e.g., low-, intermediate-, and high-risk bands) may offer more nuanced support than a single binary cut-off.

Changes in the manuscript: We have revised the paragraph in the Discussion addressing false positives to incorporate the reviewer’s concerns about sensitivity and thresholding. The updated paragraph reads: “The false positive rate (FPR) is an essential metric to evaluate the reliability of the model in clinical practice. In our study, the combined model achieved a relatively low FPR of 8.33% in the validation cohort, indicating that the model performed well in distinguishing tumor from non-tumor cases and yielding high specificity (~91.7%) alongside good overall discrimination (AUC >0.83). However, the sensitivity at the chosen operating point remained moderate (55.6%), implying a risk of false negatives (missed LNM) that warrants cautious interpretation, particularly when LNM status could alter management. This operating profile is conservative—helping to reduce unnecessary interventions and avoid over-diagnosis—yet it underscores the need to tailor decision thresholds to clinical priorities: lowering the threshold to increase sensitivity when avoiding under-treatment is critical, and raising the threshold to emphasize specificity when minimizing over-treatment is paramount. For routine use, a balanced operating point (e.g., near the Youden index) may provide an appropriate trade-off. We recommend site-specific calibration and the use of calibration assessment and decision-curve analysis to identify thresholds that maximize net benefit. Probability-based risk stratification (e.g., defining low-, intermediate-, and high-risk bands) may further support nuanced decision-making. Although some misclassifications were observed, the model demonstrated a favorable balance among sensitivity, specificity, and FPR, and is intended to complement—rather than replace—clinical judgment in predicting preoperative LNM in CRC patients.”(Lines 358–376).

Comment 4: Tumor segmentation was performed manually, while colorectal segmentation used an automated tool. Could the authors elaborate on how segmentation variability may influence radiomics feature stability, and whether robustness testing was performed?

Response: We appreciate the reviewer’s important question. Segmentation variability can affect the stability of radiomics features, particularly boundary-sensitive shape and texture descriptors. For the tumor ROI, we used venous-phase CT to enhance contrast between the tumor and surrounding tissues and performed manual delineation in ITK-SNAP with a rigorous quality-control workflow: two senior gastrointestinal radiologists reviewed and confirmed all tumor contours to ensure complete lesion coverage and exclusion of non-tumor structures (normal bowel, lymph nodes, paracolic tissue). These procedures are described in the manuscript (Lines 153–156) and were intended to minimize intra- and inter-observer variability. For the colorectal region, we employed an automated tool based on Total Segmentator and validated its agreement with physician annotations. The automated contours showed excellent concordance with expert-defined regions (ICC = 0.994), and repeated automated segmentations of the same patient’s image demonstrated high reproducibility (ICC = 0.989), as detailed in the manuscript (Lines 164–168). These findings suggest that features derived from the colorectal region are likely to be stable with respect to segmentation variability.

Changes in the manuscript: We have expanded the Discussion to further explain how segmentation variability may influence radiomics feature stability and to place our workflow and validation results in context. The added text reads: “Segmentation variability can propagate to radiomics features via boundary placement, ROI size/shape, and the inclusion or exclusion of adjacent tissues. Boundary shifts predominantly affect shape metrics (e.g., volume, surface area, sphericity/compactness), boundary-sensitive texture features (e.g., GLCM contrast/entropy, GLRLM/GLSZM nonuniformity), and high-frequency wavelet or Laplacian-of-Gaussian features, whereas global intensity histogram features may be less sensitive to small boundary changes. Partial-volume effects at the tumor–bowel interface and inadvertent inclusion of peritumoral tissues can alter heterogeneity descriptors and impact downstream model outputs. To mitigate variability, tumor ROIs were manually delineated on venous-phase CT and reviewed by two radiologists; automated colorectal segmentation based on Total Segmentator showed excellent agreement with expert annotations (ICC = 0.994) and good repeatability (ICC = 0.989), supporting the stability of features in this region. In future work, we will explore deep learning–driven tumor auto-segmentation to further reduce operator dependence and enhance cross-center stability.”(Lines 390–403).
