# Supplementary material for: Radiomics profiling combined with clinical risk factors for preoperative Lymphatic Metastasis prediction in Colorectal cancer: A multicenter study
Source: PLoS One. 2026 Jan 16;21(1):e0340352. doi: 10.1371/journal.pone.0340352 (PMC12810846; doi:10.1371/journal.pone.0340352)
Supplement: Attachment — Submitted filename: Response_to_Reviewers_auresp_4.docx [file pone.0340352.s015.docx]

**1. Were all CT scans obtained using the same scanner parameters, slice thickness, and reconstruction algorithms across centers? Variability in imaging protocols can significantly affect radiomic feature reproducibility, so this should be discussed or controlled for statistically.**

**Response:**Thank you for your feedback on our manuscript. In our study, we first performed 0-1 normalization on the extracted radiomic features to reduce variability arising from different scanning conditions. Specifically, we employed the following approach:

Purpose of 0-1 Normalization: Different scanning devices and parameters can lead to variations in the range of feature values, which can affect the assessment of tumor characteristics. 0-1 normalization effectively eliminates these differences by standardizing the scale of feature values, allowing for valid comparisons within the same range.

Method of Implementing 0-1 Normalization: We used the following formula to transform each feature value into a scale between 0 and 1:

$$\text{X}\text{=}\frac{\text{X}\text{−}\min\text{(}\text{X}\text{)}}{\max\text{(}\text{X}\text{)−}\min\text{(}\text{X}\text{)}}$$

This approach ensures that all feature values are rescaled to the range of [0, 1].

Analysis of Normalized Features: The normalized features will be used for subsequent statistical analyses. This will enable us tomore accurately evaluate the stability of features under different scanning conditions and their impact on model performance.

By implementing 0-1 normalization, we have established a solid foundation for subsequentcomparisons and analyses, ensuring the comparability and consistency of radiomic features extracted under different scanning conditions.

**Changes in the manuscript:** We have added the following text to the statistical analysis section to clarify how we addressed the variations in images caused by different scanning devices across centers:

"First, we divided the patient data into a training set and an external validation set. After extracting features using radiomic methods, we performed 0-1 normalization on all extracted features due to significant differences in the ranges of radiomic feature values resulting from various scanning devices and parameter settings. These differences could potentially impact the evaluation of tumor characteristics. This normalization process is a crucial step to ensure the comparability of features and the reliability of the analysis results."(Lines 204-209).

**2. The manuscript does not specify whether intra- and inter-observer reproducibility was tested for ROI segmentation. Radiomics models are sensitive to manual delineation; therefore,inclusion of intra-class correlation coefficient (ICC) analyses would increase confidence in feature robustness.**

**Response:** We thank the reviewer for highlighting the importance of assessing intra- and inter-observer reproducibility for ROI segmentation. In response to this valuable feedback, we conducted intra-class correlation coefficient (ICC) analyses. Specifically, we calculated the ICC for ROI segmentations performed by the same observer on the same patient one day apart, which yielded an ICC of 0.973, indicating excellent intra-observer reliability. Additionally, we assessed the ICC between two different observers segmenting the ROI for the same patient, which resulted in an ICC of 0.972, demonstrating strong inter-observer agreement.We believe that these results affirm the robustness of our ROI segmentation process and enhance the reliability of the radiomic features extracted in our study.

**Changes in the manuscript:** In the "Image Acquisition and 3D Segmentation" section, we have added the following text to explicitly report the ICC analyses:

“ To ensure consistency and accuracy, two senior gastrointestinal radiologists reviewed and confirmed all manually segmented tumor regions, verifying segmentation accuracy and ensuring that no additional structures, such as lymph nodes or paracolic tissue, were included in the tumor ROI. To evaluate the reproducibility of ROI segmentation, we conducted intra-class correlation coefficient (ICC) analyses. The ICC for segmentation performed by the same observer on the same patient one day apart was 0.973, indicating excellent intra-observer reliability. Furthermore, the ICC calculated between two different observers segmenting the same patient’s ROI was 0.972, demonstrating strong inter-observer agreement. These results further support the reliability of our radiomic feature extraction process. ” (Lines 155-164).

**3. Which radiomic features contributed most strongly to LNM prediction? Interpretation of these features in relation to tumor biology—such as heterogeneity or vascularity—would improve readability for clinicians unfamiliar with radiomics.**

**Response:** We thank the reviewer for this insightful comment. We agree that understanding which radiomic features contribute most significantly to lymph node metastasis (LNM) prediction is crucial for clinical interpretation. we have included a SHAP (SHapley Additive exPlanations) plot in our manuscript.  In our analysis, we identified two key features that played a substantial role: original_shape_Compactness from the 3D(R) imaging features and log-sigma_3_mm_3D_gldm_SmallDependenceHighGrayLevelEmphasis from the 3D(C) imaging features.

Interpretation of Key Features:

The original_shape_Compactness is indicative of the tumor's geometric properties. A higher compactness suggests a more homogeneous tumor structure, which may correlate with a lower likelihood of metastasis. In contrast, irregular shapes often reflect increased tumor heterogeneity, potentially indicating a higher risk of lymph node involvement. This relationship underscores the importance of tumor morphology in understanding metastatic behavior.

The log-sigma_3_mm_3D_gldm_SmallDependenceHighGrayLevelEmphasis feature highlights the distribution of high gray-level values within the tumor. This characteristic can signify areas of increased vascularity, which are essential for tumor growth and metastasis. Enhanced vascularity may facilitate the spread of tumor cells, thereby increasing the risk of lymph node metastasis. By elucidating these associations, we aim to provide clinicians with a clearer understanding of how specific imaging features relate to tumor biology.

**Changes in the manuscript:** We have added the following text to the Discussion to elaborate on the contributions of these features to LNM prediction:

“Based on the SHAP values obtained from our analysis, we found that the original_shape_Compactness and log-sigma_3_mm_3D_gldm_SmallDependenceHighGrayLevelEmphasis features significantly contribute to our predictive model for lymph node metastasis. The former reflects tumor geometric properties, suggesting that higher compactness may correlate with lower metastatic potential. In contrast, the latter emphasizes high gray-level areas that are indicative of increased vascularity, thus highlighting the importance of tumor biology in predicting lymph node involvement. By elucidating these associations, we aim to provide clinicians with a clearer understanding of how specific imaging features relate to tumor biology.” (Lines 333–341).

**4. Include 95% confidence intervals for AUC values.**

Thank you for your suggestion to include 95% confidence intervals (CIs) for the AUC values. We have incorporated the corresponding 95% confidence intervals for all AUC values in the manuscript, and they are highlighted in red for clarity.
